# Activation of AMPK by Bitter Melon Triterpenoids Involves CaMKKβ

**DOI:** 10.1371/journal.pone.0062309

**Published:** 2013-04-25

**Authors:** Tristan J. Iseli, Nigel Turner, Xiao-Yi Zeng, Gregory J. Cooney, Edward W. Kraegen, Sheng Yao, Yang Ye, David E. James, Ji-Ming Ye

**Affiliations:** 1 Diabetes and Obesity Research Program, Garvan Institute of Medical Research, Sydney, Australia; 2 Health Innovations Research Institute and School of Health Sciences, RMIT University, Melbourne, Australia; 3 Shanghai Institute of Materia Medica, Chinese Academy of Sciences, Shanghai, China; Virginia Commonwealth University, United States of America

## Abstract

We recently showed that bitter melon-derived triterpenoids (BMTs) activate AMPK and increase GLUT4 translocation to the plasma membrane *in vitro*, and improve glucose disposal in insulin resistant models *in vivo*. Here we interrogated the mechanism by which these novel compounds activate AMPK, a leading anti-diabetic drug target. BMTs did not activate AMPK directly in an allosteric manner as AMP or the Abbott compound (A-769662) does, nor did they activate AMPK by inhibiting cellular respiration like many commonly used anti-diabetic medications. BMTs increased AMPK activity in both L6 myotubes and LKB1-deficient HeLa cells by 20–35%. Incubation with the CaMKKβ inhibitor, STO-609, completely attenuated this effect suggesting a key role for CaMKKβ in this activation. Incubation of L6 myotubes with the calcium chelator EGTA-AM did not alter this activation suggesting that the BMT-dependent activation was Ca^2+^-independent. We therefore propose that CaMKKβ is a key upstream kinase for BMT-induced activation of AMPK.

## Introduction

Type 2 diabetes (T2D) accounts for 90% of the world’s escalating diabetes problem, with 439 million people expected to have diabetes by 2030 [1] at a predicted global healthcare cost of US $490 billion per annum [2]. However, current treatments for T2D, insulin resistance and obesity remain insufficient and often result in undesirable side-effects [3]. Traditional medicines have been an attractive source of novel anti-diabetic therapeutics. One such source is the bitter melon (BM, *Momordica charantia*) with extracts having been used to treat diabetes-like symptoms in humans for hundreds of years. Bitter melon extracts have been reported to have hypoglycaemic, hypolipidemic, antioxidant and anti-inflammatory effects and improve skeletal muscle insulin signalling *in vivo* [4]–[7]. Our recent studies have revealed that bitter melon-derived triterpenoids (BMTs) can increase fat oxidation and glucose tolerance *in vivo* and stimulate glucose transporter 4 (GLUT4) translocation via the activation of AMP-activated protein kinase (AMPK) in both muscle and fat cells [8]. Furthermore, we have recently demonstrated a sustained efficacy of a triterpenoid in eliminating hyperglycaemia in T2D mice [9]. These reports suggest that triterpenoids may be an attractive source of new anti-diabetic therapeutics via activating AMPK.

AMPK is a potential target for the treatment of T2D as activation of this kinase can promote fat oxidation to counteract insulin resistance [10]–[12]. AMPK activation is associated with the actions of anti-diabetic drugs including metformin and thiazolidinediones (TZDs, such as rosiglitazone and pioglitazone). However, there are limitations to these treatments. Metformin is not sufficient in the long term to control hyperglycemia and concerns have been raised about cardiac complications associated with TZDs [13], [14]. One common feature of metformin and rosiglitazone is that both activate AMPK indirectly by inhibiting mitochondrial Complex I [15]. A similar effect has also been found with other AMPK-activating agents such as berberines [15], [16] and resveratrol [17]. Conceivably, activation of AMPK without disturbing mitochondrial respiration may be a preferred mechanism to avoid some of the side-effects of these anti-diabetic agents [18], [19].

AMPK is a heterotrimer consisting of a catalytic α subunit, a scaffolding β subunit [10]–[12], [20] and regulatory γ subunit, which is responsible for binding adenosine nucleotides, AMP, ADP and ATP [21], [22]. AMPK is activated in response to changes in the adenylate charge [23], leading to an increase in AMP- or ADP-associated AMPK and phosphorylation by the major upstream kinases LKB1 [24] or CaMKKβ [25], [26]. LKB1-mediated activation of AMPK is dependent on binding to MO25α and STRADα, which regulates its subcellular localisation [27]. CaMKK, has two isoforms (α and β) with CaMKKβ considered the major AMPK kinase due to its ability to directly bind to AMPK which then directs its kinase activity away from its other substrates and towards AMPK [28]. Phosphorylation inhibits the autonomous activity of CaMKKβ which is relieved by Ca^2+^/CaM binding [29].

The purpose of this study was to identify the mechanism by which BMTs increase AMPK phosphorylation. Our results in the present study rule out direct allosteric activation, as well as indirect activation of AMPK through inhibition of mitochondrial respiration. We used LKB1-deficient HeLa cells together with the CaMKKβ inhibitor and calcium chelators to confirm that BMTs activate AMPK through CaMKKβ activation without altering intracellular calcium flux. These data support BMTs as being a novel class of AMPK activators and advocates the CaMKK-AMPK pathway as a potential target for novel anti-diabetic therapeutics.

## Materials and Methods

Column chromatographic separations were carried out by using silica gel H60 (300–400 mesh, Qingdao Haiyang Chemical Group Corporation, China), MCI GEL CHP20P (75–150 mm, Mitsubishi, Japan), and Sephadex LH-20 (Pharmacia Biotech AB, Sweden) as packing materials. HSGF254 silica gel TLC plates (Yantai Chemical Industrial Institute, China) were used for analytical TLC. The Analytical HPLC system was composed of Waters 2690 separations module, Waters 996 diode array detector (Waters, USA), and All-Tech 2000 ELSD. A LiChrospher 100 RP-18e column (125×4 mm i.d.; particle size 5 µm) was used for the separation. The Preparative HPLC system composed of two PrepStar SD-1 solvent delivery modules, a ProStar UV-Vis 320 detector, and a ProStar 701 Fraction Collector (Varian, USA). A LiChrospher 100 RP-18 (Merck, USA) column (220×25 mm i.d.; particle size 12 µm) was used for isolation. 5-Aminoimidazole-4-carboxamide-1-β-D-ribofuranoside (AICAR) was obtained from Toronto Research Chemicals (Ontario, Canada), STO-609 acetate was from Tocris Bioscience (Bristol, UK), EasyTide [γ-^32^P] ATP (10 µCi/ml) was from Perkin Elmer (Boston, MA, USA), AMARA peptide was from Auspep (Vic, Australia); ionomycin (calcium salt), bovine serum albumin (BSA), α-MEM, DMEM, foetal bovine serum (FBS) and 100×antibiotic/antimycotic and Pen/Strep/Glutamine (PSG) were from Invitrogen (Auckland, NZ). EGTA-AM was from Calbiochem (La Jolla, CA, USA) and Abbott compound (A-769662) was a gift from Kei Sakamoto (Dundee, UK). The pan-AMPKβ antibody was a gift from David Carling (London, UK). The 14-3-3β, CaMKI pT177 and total antibodies were from Santa Cruz Biotechnology Inc. (CA, USA); all other antibodies were from Cell Signaling Technologies (Beverly, MA, USA).

### Purification of Tetracyclic Triterpenoids from Bitter Melon

**Figure 1:**
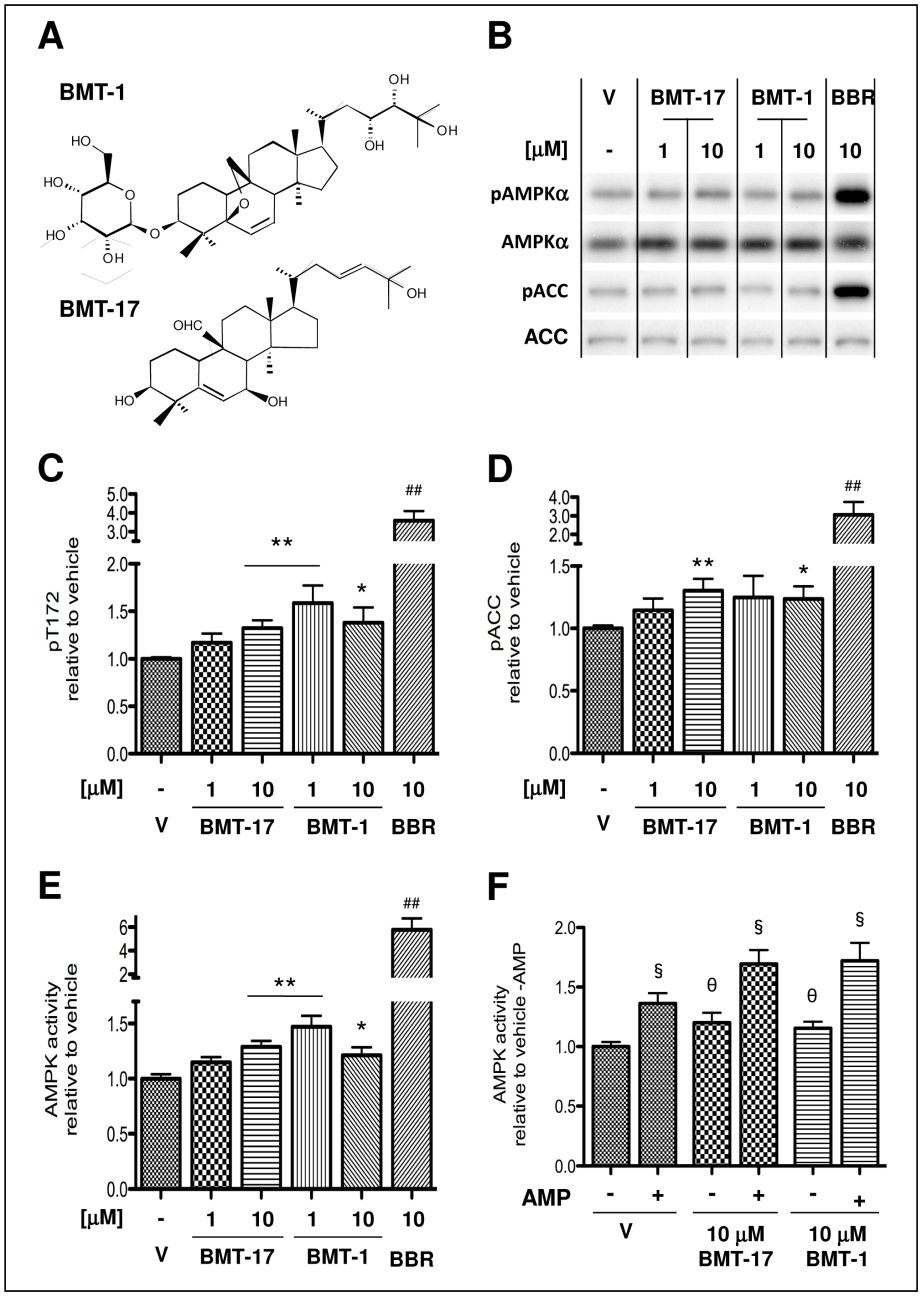
Bitter melon triterpenoids (BMT) phosphorylate and activate AMPK in L6 myotubes. Structure of BMT-1 and BMT-17 (**A**). Serum-starved L6 myotubes were treated with either vehicle (V), BMT-1 or BMT-17 at 1 or 10 µM, or 10 µM berberine, (BBR) for 30 min before lysis (**B–F**). 10 µg of lysate was analysed by Western blot analysis (**B**) with densitometric analysis plotted relative to vehicle (V) within each experiment for pT172 (**C**) and pACC (**D**). AMPK complexes were isolated from 50 µg of lysate by immunoprecipitation with a pan-AMPKβ antibody before assessment by *in vitro* AMPK assay; in the presence (both **E** and **F**), or absence (**F** only) of 200 µM AMP. Data are means ± SEM for 3–6 independent experiments each containing 2–4 repeats. *p<0.05, **p<0.01 by one-way ANOVA to V; ^##^p<0.01 to V by Student’s unpaired t-test. 1F was analysed by two-way ANOVA with θ p<0.05 for compound effects, ^§^p<0.05 for AMP effects. No interaction between treatment groups was apparent.

Purification of BMT-1 has been described previously [8]. Briefly, an initial ethanol extraction was obtained from freeze-dried bitter melon extracted with 80% aqueous ethanol, and then partitioned with dichloromethane and n-butanol, successively. The n-butanol soluble part was subjected to macroporous resin column chromatography eluting with ethanol/water mixture with various volume ratios to produce different subfractions. Based on preliminary tests in animals, one selected subfraction was then subjected to normal phase silica gel column chromatography with gradient elution (CHCl_3_: MeOH 100∶7∼100∶35) to yield 8 fractions. BMT-1 was purified from fraction 6 by repeated column chromatography over MCI gel (MeOH in water 40∼95%), silica gel (CHCl_3_: MeOH 8∶1∼8∶3), and Sephadex LH-20 (MeOH). BMT-17 was also purified from this subfraction using repeated column chromatography over silica gel (CHCl_3_: MeOH 12∶1∼5∶1), Sephadex LH-20 (CHCl_3_: MeOH 1∶1), and finally preparative HPLC (CH_3_CN in water 45∼70%, 90 min, 15 ml/min). The structure of BMT-17 was determined by NMR spectroscopic data and its purity (>98%) was determined by HPLC analysis. The structures of both compounds can be seen in Fig. 1A. BMT-1 is soluble in MeOH, pyridine and DMSO while BMT-17 is soluble in chloroform and DMSO. As such both compounds were solubilised in DMSO for this study.

### Tissue Culture, Respiration and AMPK Assays

**Figure 2:**
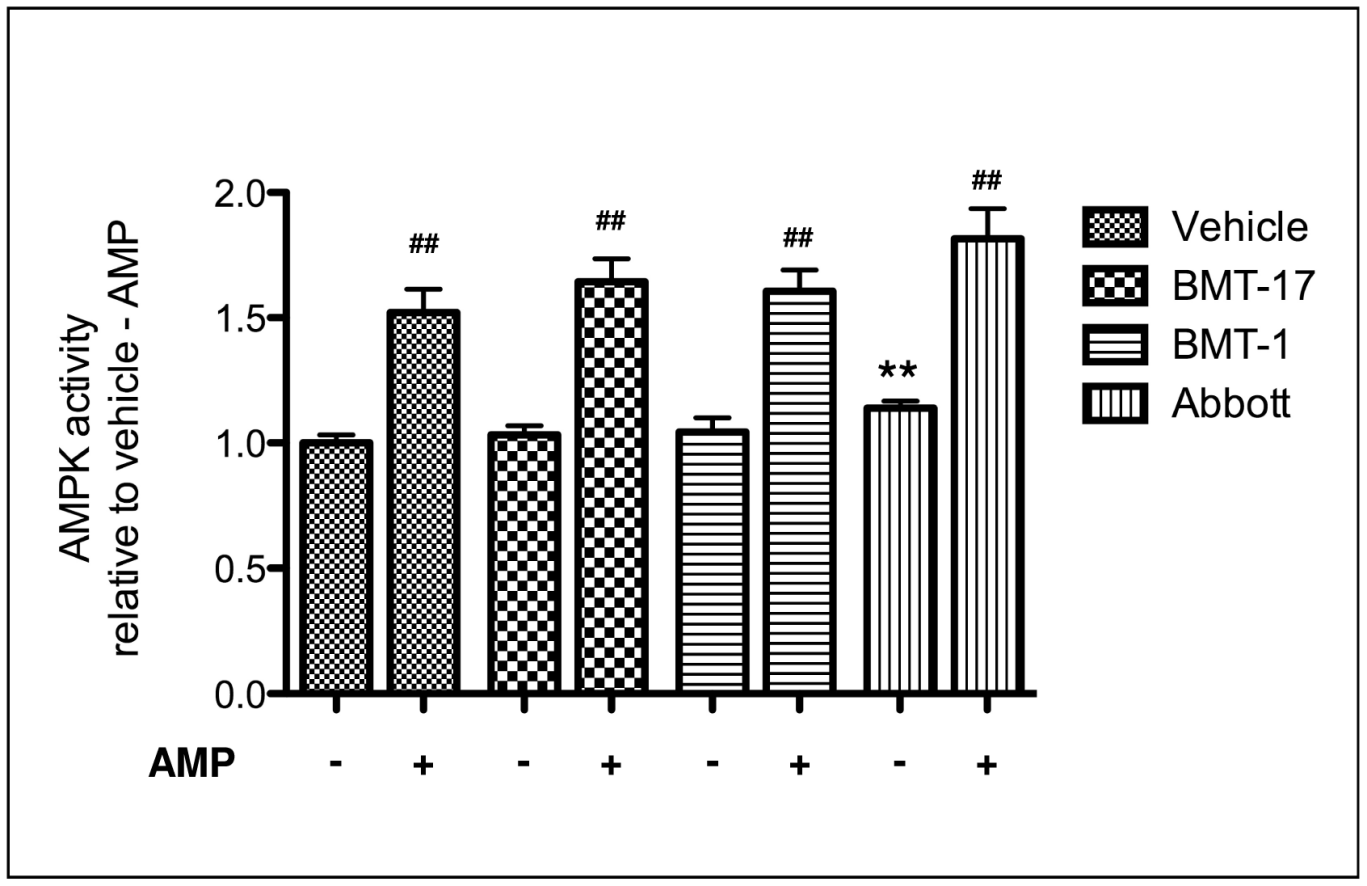
BMTs do not directly activate AMPK in isolated AMPK complexes. AMPK complexes were isolated from untreated 18 hr serum-starved L6 myotube lysates by immunoprecipitation using a pan-AMPKβ antibody. The isolated AMPK was then incubated *in vitro* with either vehicle (V) alone, 10 µM BMT-17, BMT-1 or Abbott compound (A-769662) in the presence or absence of 200 µM AMP in AMPK assay buffer for 10 min at 30°C. The *in vitro* kinase assay was then initiated with the addition of 200 µM ^32^P-Mg.ATP and assayed for a further 10 min at 30°C. Incorporation of the ^32^P into the substrate peptide was then assessed by beta scintillation counting. Data are means ± SEM for 5 separate experiments. **p<0.01 for compound effect, ^##^p<0.01 for AMP effects by two-way ANOVA. No interaction between treatment groups was apparent.

**Figure 3:**
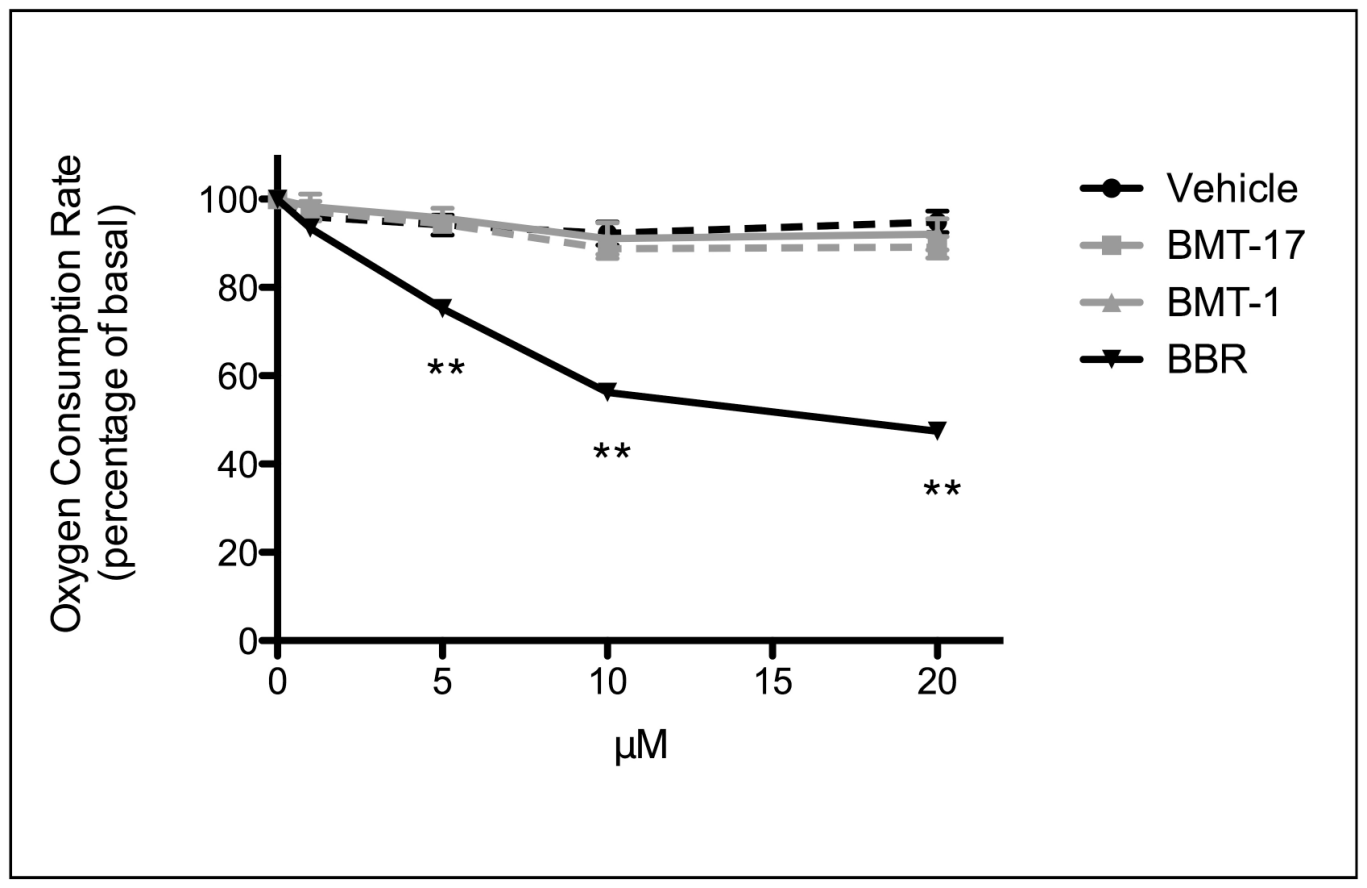
BMTs do not activate AMPK by inhibiting cellular respiration. Dose-dependent inhibition of the oxygen consumption rate in L6 myotubes by berberine (BBR, solid black line) but not by BMTs 17 (dotted grey line) or 1 (solid grey line) relative to vehicle (V) alone (DMSO, dotted black line). Oxygen consumption rates were measured in low glucose DMEM using a Seahorse XF24 extracellular flux analyser at 37°C. Data are means ± SEM (n = 7–8 per group over 2 independent experiments) and are expressed as a percentage of the basal rate (100%). **p<0.01 vs V by Student’s unpaired t-test.

**Figure 4:**
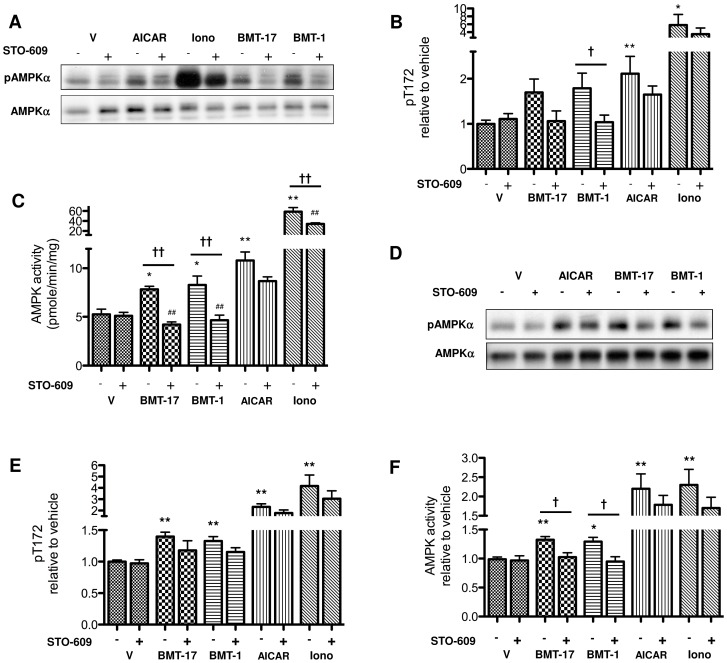
Activation of AMPK involves CaMKKβ and not LKB1. Serum-starved HeLa cells (**A–C**) or L6 myotubes (**D–F**) were pre-treated ±1 µM STO-609 for 15 min before being incubated in either vehicle (0.1% DMSO), 2 mM AICAR, or 10 µM BMTs 17 or 1, for 30 min; or 1 µM ionomycin (Iono) for 10 min before lysis. Whole cell lysates (10 µg) were analysed by Western blot analysis (**A** and **D**) and quantified by densitometry (**B** and **E**). AMPK complexes were isolated by immunoprecipitation with a pan-AMPKβ antibody and assayed by *in vitro* AMPK kinase assay (**C** and **F**). Incorporation of ^32^P into the AMARA substrate peptide was then assessed by β–scintillation counting. Data are means ± SEM (n = 4–8 per group). *p<0.05, **p<0.01 for compound effects by two-way ANOVA; ^##^p<0.01 for STO-609 effects, ^†^p<0.05, ^††^p<0.01 for interactions between treatment groups.

**Figure 5:**
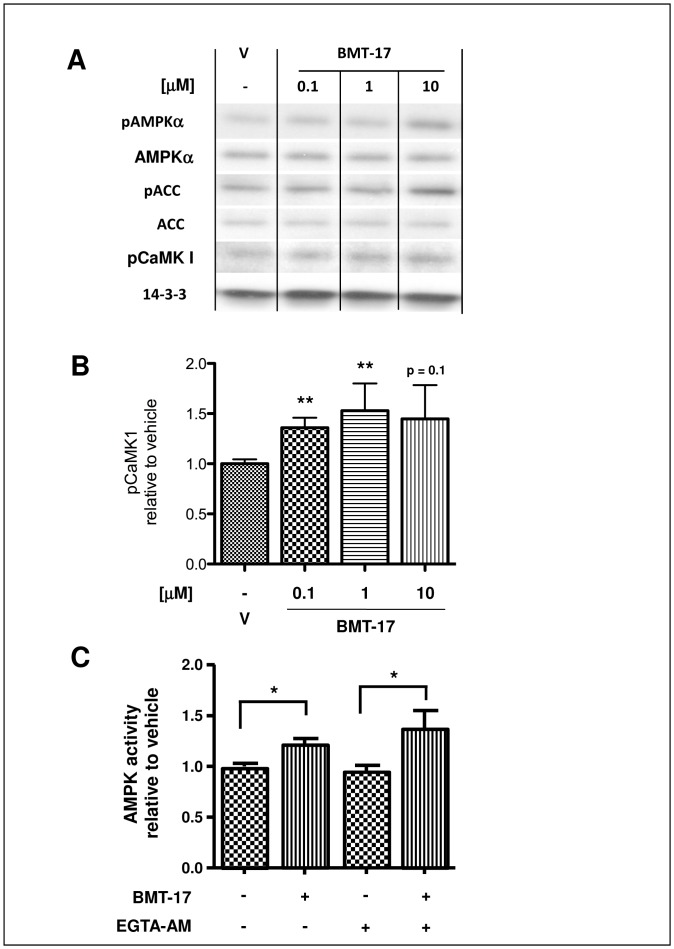
BMTs activate AMPK through CaMKK and not through intracellular Ca^2+^ changes. Serum-starved L6 myotubes were treated with either vehicle (V) alone, BMT-17 at 0.1 µM, 1 µM or 10 µM for 30 min before lysis; 10 µg of lysate was then analysed by Western blot (**A**) and quantified by densitometry (**B**). A representative blot is shown. Serum-starved L6 myotubes were pre-treated with either 150 µM EGTA-AM (+) or diluent (-) for 15 min before then being treated with 10 µM BMT-17 or vehicle for a further 30 min before lysis. AMPK was then isolated from 50 µg lysate by pan-AMPKβ immunoprecipitation and assessed by AMPK *in vitro* kinase assay (**C**). Data are means relative to untreated vehicle control (V) ± SEM from 5 independent experiments. *p<0.05, **p<0.01 to V by one-way ANOVA.

L6 myoblasts (up to passage 17) were cultured in α-MEM supplemented with 10% heat-inactivated FBS and 1% antibiotic/antimycotic. HeLa cells (ATCC) were cultured in DMEM (high glucose) supplemented with 10% heat-inactivated FBS, 1×PSG (Gibco, Invitrogen). Both were kept at 37°C in a humidified 5% CO_2_ incubator. For differentiation into myotubes, L6 cells were cultured in α-MEM supplemented with 2% heat-inactivated FBS and 1% antibiotic/antimycotic for 5–7 days before interventions. Before acute treatments, cells were moved into serum-free media for 18 hrs. Cells were then treated with either vehicle (0.1% DMSO), 2 mM AICAR, or BMTs, at concentrations indicated in the figures 1, 2, 3, 4, 5, for 30 min. As 1 µM ionomycin was toxic to the cells after 30 min, we incubated cells with ionomycin for 10 min. Cells were then quickly washed twice in ice-cold PBS before addition of lysis buffer (50 mM HEPES pH 7.4, 150 mM NaCl, 1% Triton X-100, 1 mM EDTA, 1 mM EGTA, 10% glycerol, 1 mM DTT) containing phosphatase inhibitors (40 mM NaF, 4 mM Na_4_P_2_O_7_, 2 mM Na_3_VO_4_) and 1× Complete Protease Inhibitor Cocktail (Roche Applied Sciences, Indianapolis, IN, USA). Lysates were then snap-frozen in liquid nitrogen and stored at −80°C before use. Whole cell lysates were prepared by clearing cell debris by centrifugation before quantification of protein content by BCA (Pierce Technology, Rockford, IL, USA) or Bradford (Bio-Rad Laboratories, Hercules, CA, USA) protein assay.

For respiration experiments, L6 myotubes were cultured and differentiated in an XF24 culture plate (Seahorse Bioscience, Billerica, MA, USA). Differentiated L6 myotubes were then serum-starved 18 hrs in unbuffered α-MEM pH 7.4 (Invitrogen Cat No. 12000-022). Basal oxygen consumption rates (OCR) were measured in triplicate cycles of mix (3 min), wait (2 min) and measure (3 min). Repeated triplicate cycles were performed following each consecutive dose-dependent addition of vehicle, BMT-1, BMT-17 or berberine. For quality control, the OCRs were compared to those of untreated myotubes over time and total protein content of each well following the final measurement. To normalise well-well variation, the basal OCR of each well was set to 100% and the dose-dependent effects of each injection were compared to this.

For *in vitro* AMPK assays, AMPK complexes were first isolated by immunoprecipitation. Briefly, 50 µg of lysate was combined with 0.5 µl anti-pan-AMPKβ antibody (to ensure all complexes were retained), 10 µl Protein A/G Sepharose (Amersham) and 700 µl of lysis buffer in the absence of protease inhibitor cocktail and incubated rotating at 4°C for 2 h. The beads were then washed in 700 µl lysis buffer, then in 700 µl HGE buffer (50 mM Hepes pH 7.4, 10% glycerol, 0.1 mM EDTA) and excess fluid was removed using a 30 G needle. The isolated AMPK was then incubated in assay buffer (50 mM HEPES pH 7.4, 5% glycerol, 5 mM MgCl_2_ and 200 µM AMARA peptide) in the absence or presence of 200 µM AMP. Assays were initiated by addition of ^32^P-ATP (200 cpm/pmole) at 30°C for 10–14 min. An aliquot of each sample was spotted onto P81 paper and stopped in 1% phosphoric acid. Incorporation of ^32^P into the AMARA peptide was assessed by β-scintillation counter.

### Western Blot Analysis

After protein quantification, whole cell lysates were reduced in Laemmli buffer containing 15 mM DTT at 65°C for 10 mins. Lysates (10 µg of total protein) were then subjected to SDS-PAGE on 9% gels and transferred onto Hybond PVDF membranes (GE Healthcare, Buckinghamshire, UK) overnight at a constant current (40 mA) in Tris/Glycine transfer buffer containing 10% methanol. Membranes were then blocked in 2% BSA in TBS containing 0.1% Tween 20 (TBST) for at least 1 hr before being incubated in rabbit polyclonal antibodies overnight (∼16 hrs) at 4°C. Membranes were then washed in TBST and then incubated in HRP-conjugated donkey anti rabbit secondary antibody (Jackson Immuno Research Laboratories, West Grove, PA, USA) for 1–2 hrs at room temperature. Membranes were then washed in TBST and proteins were visualised by ECL (PerkinElmer, Waltham, USA) and exposed on X-ray film before scanning and densitometric analysis using ImageJ (NIH, Bethesda, MD, USA).

### Statistical Analysis

Results are presented as means ± SEM. When multiple doses were used, individual compounds were compared to vehicle control by one-way ANOVA using a Fisher’s Least Significant Difference *post-hoc* test. When two separate treatments were considered, individual two-way ANOVA analyses were used to assess treatment effects and interactions between treatment effects, followed by post hoc tests to compare each group to the vehicle control group. An unpaired Student’s t-test was used to assess statistical significance where one group was compared to vehicle control. For each statistical consideration, p≤0.05 was taken to indicate a significant difference.

## Results

### BMTs Increase AMPK Phosphorylation and Activity

Initially we confirmed that the lead candidate from the original study, BMT-1 [8], and a structurally similar but lower molecular weight compound, BMT-17 (Fig. 1A), resulted in AMPK phosphorylation in L6 myotubes. At the same time we assessed if this increase in AMPK phosphorylation led to phosphorylation of the key AMPK substrate ACC at Ser-79. Incubation of serum-starved L6 myotubes with 1 µM and 10 µM of these BMTs increased both AMPK Thr-172 and ACC Ser-79 phosphorylation, though not to the extent of the positive control berberine (Fig. 1B–D). To assess if these BMTs increased *in vitro* AMPK activity, we isolated AMPK complexes from these lysates using a pan-AMPKβ antibody that recognizes the conserved C-terminus of both AMPKβ subunits essential for complex formation [30]. Both BMTs increased AMPK activity (15–50%) though, again, not to the extent of the positive control berberine (Fig. 1E). BMTs activated AMPK both in the absence and presence of the allosteric activator, AMP, to a similar degree. However, no interaction was evident with a two-way ANOVA (Fig. 1F) indicating that this activation was independent of and additive to AMP-dependent activation.

### BMTs do not Allosterically Activate AMPK

To assess if these BMTs could directly activate AMPK *in vitro*, in an allosteric manner similar to AMP or to the recently described compound A-769662 [31], we again isolated AMPK from untreated serum-starved L6 myotubes. The isolated AMPK was then incubated *in vitro* with 10 µM BMT-1 or BMT-17 or Abbott compound A-769662 in the presence or absence of 200 µM AMP for 10 min at 30°C before initiation of the *in vitro* AMPK assay with the addition of ^32^P.MgATP (Fig. 2). Unlike the allosteric activators AMP or A-769662, neither BMT-1 nor -17 could increase AMPK activity *in vitro,* nor could they enhance the AMP-dependent activation of the complex. The reason why the Abbott compound (A-769662) only mildly increased AMPK activation may relate to the fact that L6 myotubes are derived from rat skeletal muscle which should predominantly express α2β2-containing AMPK complexes, which are more AMP- and less Abbott compound-sensitive [32], [33]. Furthermore, AMPK has been shown to be both nucleotide- and Abbott compound-insensitive with increased basal activity [11], [32], which may also contribute to the subtle increase in AMPK activity seen here. Together, these data suggest that BMTs do not activate AMPK in an allosteric manner similar to either AMP or the Abbott compound, nor do they enhance AMP-dependence of the AMPK complex.

### BMTs do not Activate AMPK by Altering Cellular Respiration

As BMTs did not activate AMPK directly, indirect mechanisms for AMPK activation were investigated. We recently showed that metformin, rosiglitazone and another natural product, berberine, could activate AMPK by altering the cellular energy charge through inhibiting Complex I of the mitochondrial respiratory chain [15]. We therefore assessed the effect of BMTs on whole cell respiration in L6 myotubes using a Seahorse Bioscience XF24 Extracellular Flux Analyzer (USA). BMTs did not affect whole cell respiration, in contrast to berberine, which induced a dose-dependent inhibition of respiration (Fig. 3). These data were consistent with respiration experiments performed on isolated mouse skeletal muscle mitochondria (data not shown). Together these results indicate that unlike many commonly used anti-diabetic therapeutic agents, BMTs do not activate AMPK by altering cellular respiration.

### BMTs Activate AMPK through CaMKKβand not through LKB1

As AMPK is phosphorylated and activated by AMPKK-dependent phosphorylation, we assessed the involvement of the AMPKKs in the BMT-dependent AMPK activation. The predominant AMPKKs are LKB1 and CaMKKβ. To evaluate the involvement of LKB1, we assessed the ability of these BMTs to increase AMPK phosphorylation and activity in LKB1-deficient HeLa cells [24]. Both BMT-17 and BMT-1 acutely phosphorylated and activated AMPK in HeLa cells, suggesting that LKB1 is not necessary for these BMT-dependent effects (Fig. 4A–C). To assess if CaMKKβ itself was responsible for the increase in AMPK phosphorylation and activity, we pre-incubated the HeLa cells with 1 µM of the cell-permeable CaMKK inhibitor, STO-609, for 15 min before incubation with the BMTs. Pre-incubation with STO-609 completely blocked the BMT-dependent AMPK phosphorylation and activation confirming that CaMKKβ activity was essential for the BMT-dependent AMPK activation. The partial inhibition of the ionomycin-dependent AMPK activation by STO-609 is consistent with previous reports under these conditions [26], [34], while the minimal effects on AICAR-dependent activation can be attributed to STO-609 only attenuating one of the multiple actions of AMP on AMPK activation [35]. Together this suggests that activation of AMPK by BMTs involves CaMKKβ.

As HeLa cells lack LKB1, we therefore investigated if inhibition of CaMKKβ could attenuate the BMT-dependent increase in AMPK phosphorylation and activation in L6 myotubes, which contain LKB1. Accordingly, L6 myotubes were pre-incubated with or without 1 µM of the CaMKKβ inhibitor STO-609 for 15 min before incubation with BMTs. As with the HeLa cells, BMTs increased AMPK phosphorylation and activation and this effect was significantly inhibited by STO-609 (Fig. 4D–F). Collectively these findings indicate that BMTs activate AMPK through CaMKKβ and not through LKB1.

### BMTs Activate CaMKKβ in a Calcium-independent Manner

To assess if the BMTs increased CaMKKβ activity *per se*, we investigated if BMTs could also increase phosphorylation of the well-established CaMKKβ substrate, CaMKI, at Thr-177. Accordingly, L6 myotubes were incubated with increasing doses of BMT-17 for 30 min before analysis by Western blot (Fig. 5A and B). Indeed, BMT-17 dose-dependently increased CaMKI Thr-177 phosphorylation along with AMPK and ACC phosphorylation, indicating that BMTs activate AMPK by activating CaMKKβ.

As CaMKKβ is regulated in part by Ca^2+^/CaM binding, we then assessed whether BMTs activate CaMKKβ by increasing intracellular calcium levels. To do this L6 myotubes were pre-incubated with or without 150 µM of the cell-permeable calcium chelator, EGTA-AM, for 15 min before addition of BMT-17 and assessment of AMPK activity. Accordingly, incubation of L6 myotubes with BMT-17 significantly increased the AMPK activity. Pre-incubation with EGTA-AM to deplete the intracellular calcium levels did not alter the BMT-17-induced AMPK activation (Fig. 5C). Together these data suggest that BMTs activate AMPK through CaMKKβ in a Ca^2+^-independent manner.

## Discussion

**Figure 6:**
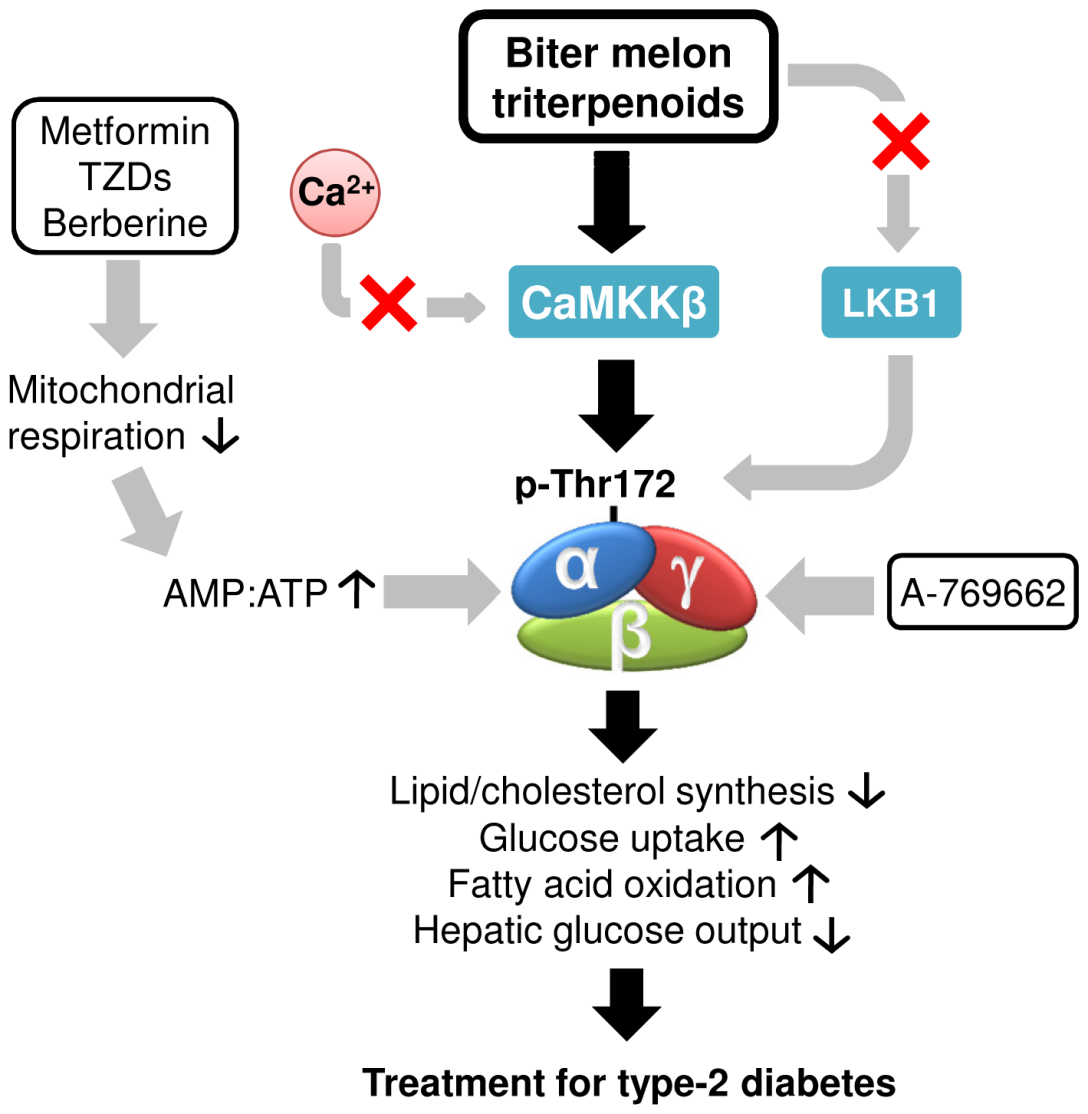
Illustration of the activation of AMPK by BMTs. BMTs activate AMPK via CaMKKβ independent of LKB1 and mitochondrial respiration. This mechanism is different from those of other anti-diabetic small molecules like metformin, TZDs, berberine or Abbott Compound (A-769662).

Here we have interrogated the mechanism by which two lead BMTs (1 and 17) phosphorylate and activate AMPK. Unlike AMP and the Abbott compound A-769662, BMTs do not allosterically activate AMPK and, unlike berberine and metformin, BMTs do not alter cellular respiration. Use of the cell permeable CaMKKβ inhibitor STO-609 on both LKB1-deficient HeLa cells and L6 myotubes has demonstrated the key role of CaMKKβ in this event (Fig. 6). We therefore suggest that BMTs activate AMPK in a distinct manner from other anti-diabetic medications.

BMTs increased AMPK activity in the absence and presence of AMP in an additive but not synergistic way suggesting the activation of AMPK by BMTs involves a mechanism distinct from that of AMP. Furthermore, unlike A-769662 and the allosteric activator AMP, neither of the BMTs could activate AMPK *in vitro* suggesting that the BMTs do not activate AMPK in a direct manner. This finding alone is an attractive attribute in an anti-diabetic therapy, as this would allow AMPK to remain responsive to other physiological stimuli such as an exercise bout.

Metformin, rosiglitazone, berberine and resveratrol all activate AMPK at least in part by inhibiting mitochondrial respiration [15], [17]. This results in an increase in the cytosolic AMP:ATP ratio, leading to an indirect activation of AMPK. Conceivably, the inhibition of mitochondrial respiration may account for some of the undesirable side-effects associated with indirect AMPK activation including lactic acidosis as a result of the associated increase in glycolysis [18], [19]. Conversely, BMTs do not activate AMPK by inhibiting mitochondrial or whole cell respiration and therefore may be more tolerated as anti-diabetic medications.

AMPK is also activated by phosphorylation by the major AMPKKs, LKB1 and CaMKKβ. Using LKB1-deficient HeLa cells and LKB1-proficient L6 myotubes together with the cell-permeable CaMKKβ inhibitor STO-609, we provide evidence to indicate that BMTs activate AMPK through CaMKKβ and not LKB1. As BMTs also increased phosphorylation of another CaMKKβ substrate, CaMKI, this indicates that BMTs may activate CaMKKβ itself and provides further evidence that BMTs are unlikely to activate AMPK through a direct interaction. Since binding to AMPK directs the CaMKKβ activity away from its other substrates CaMKI and CaMKIV [36], it is therefore also unlikely that BMTs enhance the AMPK:CaMKKβ interaction. As CaMKKβ and its substrate CaMKI are both activated by Ca^2+^/CaM binding [37], we then used the cell-permeable calcium chelator EGTA-AM to examine if Ca^2+^/CaM binding was involved in the activation of AMPK by BMTs. The inability of EGTA-AM to block the BMT-dependent AMPK activation demonstrated that the BMT-dependent AMPK activation was independent of intracellular calcium levels. Conceptually, the BMTs could be binding directly to the Ca^2+^/CaM binding site and thereby enhancing CaMKKβ activity. However, further experiments are needed to confirm this hypothesis. CaMKII, another Ca^2+^/CaM sensitive kinase can be activated by methionine oxidation or auto-phosphorylation which then maintains the protein in an active conformation [38]. CaMKKβ too can be phosphorylated at its N-terminus by CDK5 and GSK3, which regulates its Ca^2+^/CaM-independent autonomous activity [39]. This phosphorylation event is inhibitory, however it can be overcome by Ca^2+^/CaM binding. Therefore, BMTs could be increasing CaMKKβ activity by inhibiting phosphorylation by these proteins.

It is also unclear whether these BMTs can enter the cell to interact with CaMKKβ. BMTs could, in fact, signal via surface receptors to increase CaMKKβ activity. Indeed, bitter melon extracts have been shown to be estrogen receptor (ER) agonists [40], with activation increasing CaMKK and AMPK activities [41]. Accordingly, agonism of the membrane bound ER homolog GPR30 [42], a G-protein coupled receptor homolog, showed anti-diabetic properties [43]. Alternatively, as BMTs are structurally similar to bile acids [44], they could agonise the bile acid receptor TGR5, another G-protein coupled receptor which has anti-diabetic properties, though this has not been linked to CaMKKβ or AMPK activities [45]. It remains to be clarified whether BMTs may signal through surface receptors.

Natural products may be metabolised by specialized enzymes of living organisms to produce pharmacologically active metabolites. Due to the limited availability of BMTs, we were unable to investigate this possibility. However, our previous study showed that a number of cucurbitane-type triterpenes exert similar biological activity in stimulating GLUT4 translocation in L6 myotubes via activation of AMPK [8]. This suggests that BMT-1 is likely to be the bioactive compound by itself rather than serving as a precursor of an active metabolite to produce the effects as observed in the present study.

In summary, our data indicate that BMTs activate AMPK in a manner distinct from other anti-diabetic small molecules (illustrated in Fig. 6). Our findings suggest that BMTs activate AMPK through CaMKKβ activation, in a calcium-independent manner, and not through direct effects on AMPK, nor by inhibiting mitochondrial respiration. Although CaMKKβ is expressed in tissues important to glucose and lipid metabolism, such as skeletal muscle, liver and adipose [46], the role of CaMKKβ in the onset and treatment of insulin resistance and T2D is yet to be fully explored. By showing that BMTs increase AMPK activity through CaMKKβ, we provide a proof-of-concept for triterpenoids, which are abundant in the plant kingdom [44], as an attractive source of novel AMPK activators with anti-diabetic efficacy mediated by the upstream kinase CaMKKβ.
